# Bone regeneration with adipose derived stem cells in a rabbit model

**DOI:** 10.7555/JBR.32.20160066

**Published:** 2017-04-16

**Authors:** Michele Maglione, Enrico Salvador, Maria E. Ruaro, Mauro Melato, Giuliana Tromba, Daniele Angerame, Lorenzo Bevilacqua

**Affiliations:** 1. Department of Medical Sciences, University of Trieste, Trieste 34125, Italy; 2. SISSA-International School for Advanced Studies, Trieste 34136, Italy; 3. Department of Pathology and Legal Medicine, University of Trieste, Trieste 34125, Italy; 4. Elettra-Sincrotrone Trieste S.C.p.A., Trieste 34149, Italy.

**Keywords:** bone regeneration, regenerative medicine, stem cells, tissue engineering

## Abstract

It has been shown that stem cells are able to calcify both *in vitro* and *in vivo* once implanted under the skin, if conveniently differentiated. Nowadays, however, a study on their efficiency in osseous regeneration does not exist in scientific literature and this very task is the real aim of the present experimentation. Five different defects of 6 mm in diameter and 2 mm in depth were created in the calvaria of 8 white New Zealand rabbits. Four defects were regenerated using 2 different conveniently modified scaffolds (Bio-Oss® Block and Bio-Oss Collagen®, Geistlich), with and without the aid of stem cells. After the insertion, the part was covered with a collagen membrane fixed by 5 modified titan pins (Altapin®). The defect in the front was left empty on purpose as an internal control to each animal. Two animals were sacrificed respectively after 2, 4, 6, 10 weeks. The samples were evaluated with micro-CT and histological analysis. Micro-CT analysis revealed that the quantity of new bone for samples with Bio-Oss® Block and stem cells was higher than for samples with Bio-Oss® Block alone. Histological analysis showed that regeneration occurred in an optimal way in every sample treated with scaffolds. The findings indicated that the use of adult stem cells combined with scaffolds accelerated some steps in normal osseous regeneration.

## Introduction

The repair of bone defects continues to be a challenging part of many reconstructive procedures^[1–2]^. Although autogenous bone grafts remain the standard in the reconstruction of bone defects, they have disadvantages, including the limited amount of available bone and morbidity of the donor site^[3]^.

A previous approach to this problem focused on the development of various artificial materials instead of autogenous bone. However, artificial bone substitutes may expose the patient to the risks of foreign body reactions and infections^[4]^.

Recent advances in cell culture techniques may provide an elegant solution to these restrictions^[5]^. Several recent studies have reported the ubiquitous distribution of adult stem cells in various tissues and organs, including bone marrow, muscle, brain, skin, and more recently, subcutaneous fat. Stem cells represent the new frontier in the field of regenerative medicine and are seen as a promising and suitable means to overcome the mentioned drawbacks^[6]^.

Several studies report that adult stem cells can be isolated from many organs and tissues^[2]^. In particular, adipose tissue contains cells that have the ability to proliferate and differentiate into multiple cell lines^[1,7]^. These stem cells may have important applications in tissue engineering. As a matter of fact, adipose tissue-derived stem cells (ADSCs) have the potential to differentiate into bone, cartilage, fat, myocardium, skin, and neurons^[8–9]^. In current clinical practice, mesenchymal stem cells are commonly collected from the bone marrow. However, no significant differences between adipose-derived stem cells and bone marrow-derived mesenchymal stem cells from the same patient were observed with regard to the yield of adherent cells, their growth kinetics, cell senescence, differentiation capacity, and gene transduction efficiency^[10–11]^. Moreover, adipose tissue can be collected under local anesthesia more easily than bone marrow, making the procedure less invasive to the donor^[12–13]^.

The aim of this study was to investigate the differences *in vivo* between traditional bone regeneration and the combination with tissue engineering in the animal model.

## Materials and methods

Eight New Zealand rabbits weighing about 4.5 kg, treated according to the "European conventions for the protection of vertebrate animals used for experimental and other scientific purposes" (1999/575/EC) and Italian regulations (DL 116.1993), underwent the first surgery for the removal of adipose tissue. The animals were operated under anesthesia with Xylazine and Zoletil ®. After shaving and disinfecting the skin with Betadine ®, a flap was created for the removal of intrascapular adipose tissue. A single withdrawal in each animal was made from their adipose tissue and stem cells were subsequently isolated to avoid problems of rejection and the inconvenience of intervening in immunosuppressed animals. In this way any replanting will be possible with self cells taken directly from the animal. After collection, Vicryl ® sutures and an additional Betadine ® disinfection on the skin were performed. In the following days, the animals were given an antibiotic and anti-inflammatory analgesic therapy with enrofloxacin (Baytril ®) and carprofen (Rimadyl ®) to prevent complications.

### Isolation of adult mesenchymal stem cells 

The removed adipose tissue was transported in the laboratories of SISSA of Trieste, where the process of isolation of mesenchymal stem cells immediately began. The extracellular matrix was digested by a 0.1% collagenase solution in a water bath at 37 °C for 60 minutes. Thereafter, the cells obtained were seeded in Dulbecco's modified Eagle's medium containing 10% bovine serum and antibiotics (control medium) and centrifuged for 3 minutes at 1,500 r/minute. Then, yjey were filtered through a nylon membrane with a pore size of 100 µm and the cells were placed in control medium culture. The selection of cells that adhered to diskette and the gradual elimination of adipocytes were made following a well described protocol by Rietze *et al.*^[14]^.

### Preparation of scaffolds

To support cell growth in the plant site we decided to use two different scaffolds produced by Geitslich ®: deproteinized bovine bone (Bio-Oss ® Block Geistlich) and bovine cancellous granular with the addition of a collagen matrix to 10% (Bio-Oss Collagen ® Gei-stlich). The Bio-Oss ® Block was processed under sterile conditions to obtain discs with a diameter of5 mm and a thickness of 2 mm so that it fits perfectly with the type of defect.

The Bio-Oss Collagen ® were cut in two portions, since preliminary experiments suggested that this was the amount needed to adequately fill the defect.

### Osteogenic differentiation

For osteogenic differentiation, it was decided to adopt a protocol developed by Kakudo, which was already well documented. Cells differentiated in this way are able to calcify very quickly if implanted subcutaneously, but there is no evidence in the literature on the present operation to repair a critical defect. A total of 1,000,000 cells were seeded in each scaffold in control medium and kept for 24 hours. Thereafter, the medium was replaced with osteogenic medium, obtained by adding to the control medium 10 nmol/L dexamethasone, 10 mmol/L of β-glycerophosphate 82 g/mL acorbato-2 phosphate, and cells were allowed for differentiation for 14 days. The osteogenic medium was replaced 2 times every 7 days for a total of four substitutions.

### Creating experimental bone defect and plant

The second surgery mode of sedation and anesthesia were the same as the first. The flap was prepared for skeletonization of the parietal bones of the calvaria of rabbits. We proceeded to create five experimental bone defects (two on each parietal bone and one before that) of 6 mm in diameter and 2 mm in depth. Defects can be produced with standardized trephine burs, using continuous saline irrigation for cooling^[15]^. Using a special caliber created for the experiment, we checked the precise size of defects before insertion of the scaffolds alone and enriched with cells differentiated into the osteogenic line.

Defects were grafted differently: on the right of the rabbit head, with Bio-Oss ® Block and mesenchymal stem cells in the caudal defect and with Bio-Oss Collagen ® and mesenchymal stem cells in the cranial one; on the left, Bio-Oss ® Block in the caudal defect and Bio-Oss Collagen ® in the cranial one, both without the addition of stem cells. The fifth defect, the frontal one, was left empty and used as internal control in each animal ( ***Supplementary Fig.1***, available online). The defects filled with different materials were coated with a collagen membrane (BioGide Geistlich ®) fixed with 5 specially modified titanium pins (Altapin ®).

Finally, the flaps were sutured by wire Vicryl ® 3/0 and steel clips were applied outside on the skin, preventing the reopening of the flap. The skin was disinfected again with Betadine ® and animals were treated with antibiotic (enrofloxacin, Baytril ®), and anti-inflammatory/analgesic (car-profen, Rimadyl ®) therapy. No animal showed signs of suffering during the postoperative period, tightly controlled and recorded in audiovisual behavior. Thanks to the developed surgical technique, it was not necessary to complete any sacrifice before the scheduled date.

### Sacrifice and sampling

Two animals were sacrificed by intravenous injection of Tanax ® after general anesthesia with Zoletil ® respectively at 2, 4, 6 and 10 weeks. The bone samples, taken immediately after sacrifice, were immersed in 4% buffered formaldehyde. Every sample was subjected to Micro-CT scan and then to histological analysis.

### Preparation of samples

The marked samples were immersed in a solution of 40% formic acid and formate buffer in a 1:1 ratio for 5 days, long enough to ensure adequate decalcification. Samples were then post-fixed overnight in 10% buffered formalin, dehydrated through an ascending scale of alcohols (from 50% to 100%), clarified with xylene and fixed with permeating liquid paraffin at 60 °C. The material was then embedded in paraffin solidified at room temperature, so it was possible to obtain histological sections 5–10 µm thick, spread on glass slide. The sections were stained with hematoxylin to highlight nucleus and eosin for intra- and extra-cellular structures.

### Micro-CT data analysis

X-ray microcomputed tomography (µ-CT) of samples was obtained by means of a cone-beam system called TOMOLAB (www.elettra.trieste.it/Labs/TOMOLAB). The device is equipped with a sealed microfocus X-ray tube, which guaranteed a focal spot size of 5 µm in an energy range from 40 up to 130 kV, and a maximum current of 300 µA. As a detector, a CCD digital camera was used with a 49.9 mm×33.2 mm field of view and a pixel size of 12.5 µm×12.5 µm. The samples were positioned onto the turn-table of the instrument and acquisitions were performed with the following parameters: distance source-sample (FOD) 100 mm; distance source-detector (FDD) 200 mm; magnification 2×; binning 2×2; resolution 12.5 µm; tomographies dimensions (pixels) 1,984×1,024; slices dimensions (pixels) 1,984×1,984; number of tomographies 1,440; number of slices 864; E=40 kV, I=200 µA; exposure time 2.5 seconds. The slices reconstruction process achieved by means of commercial software (Cobra Exxim) started once the tomographic scan was completed and all the projections were transferred to the workstation. Input projections and output slices were represented by files (one file per projection and one file per slice) using arrays of 16-bit integers. Three-dimensional visualizations of the reconstructed slices were performed by means of OsiriX v.3.9.4. 64bit Imaging Software. This software allowed identification of the correct angulation and segmentation of the samples from which the planar view was extracted. From the planar view, the percentage of newly formed bone was calculated as the rate between the volume of the newly formed bone on the volume of the original defect. Calculation was performed by means of Image ProPlus 6.2 software. The image analysis procedure was assisted by a surgeon, expert of radiographies, to identify the orientation and the correct border between the newly formed bone and the native bone ( ***Supplementary Fig. 2***, available online). Through the use of a code written in IDL, we cored three volumes of 128×128×36 voxels (the first on the original bone structure adjacent to the defect, the second on the scaffold and the third at the interface between the two), binarized by Otsu algorithm for evaluation of class separability threshold^[16]^ implemented in software PORE3D.

Later, through the program GEHC MicroView, selected volumes of interest were analyzed by the stereological parameters, using Euler number ( *e*) as a selected parameter. *e* is a direct index of trabecular connectivity and can be defined as the maximum number of portions removable from the structure without losing its integrity. *e* was normalized for the parameter BVTV (bone volume/total volume)^[17–19]^

### Histological analysis

Immediately after µ-CT scans, the samples were sent to the Department of Pathology of the Hospital of Monfalcone (Gorizia, Italy) to perform histological analysis. Samples were immersed in a solution of EDTA (ethylenediaminetetraacetic acid) disodium in acid buffer for five hours. Samples were then placed in histology cassettes properly oriented. The biocassettes were then included in the histoprocessor where, with a predetermined sequence and timing, the samples were post-fixed in 10% buffered neutral formalin, dehydrated through an ascending scale of ethanol (from 50% to 100%), clarified with xylene and permeated by liquid paraffin at 60 °C. The material was then embedded in paraffin and allowed to solidify on chilled plates. The obtained block was then sectioned by a microtome and 8 µm thick histological sections were spread on a glass slide and placed in an oven at 60 °C for 1 hour to ensure a good adhesion of the sections to the glass slides and at the same time to dissolve the paraffin excess. Subsequently, the sections were stained with hematoxylin and eosin. After the staining, the preparations were dehydrated and mounted with resin, which was placed on the coverslip. During histological analysis of the samples, we focused on the following fundamental aspects:

- Comparison between the ossification with and without matching mesenchymal stem cells to two different scaffolds;

- Comparison of the integration of the scaffolds in the context of the newly formed tissue, with and without using mesenchymal stem cells.

## Results

### Micro-CT data results

**Fig.1:**
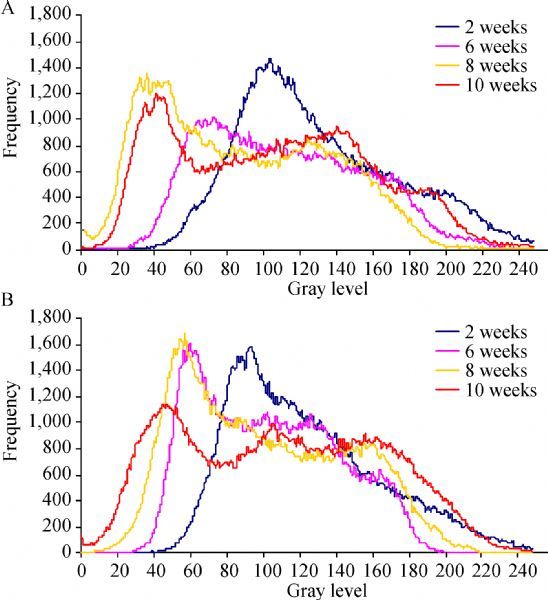
Comparison of pixel frequency of gray level in the samples treated with Bio-Oss without stem cells (A) or Bio-Oss and stem cells (B) at different weeks.

Similar findings were registered in all the analyzed samples: the quantity of new bone (higher gray levels) showed a trend to increase with the animals’ age. When comparing the graphic curves obtained for both Bio-Oss ® and Bio-Oss ® with mesenchymal stem cells, the quantity of new bone for the Bio-Oss ® with mesenchymal stem cells samples was higher when age was considered. In particular, the 10-week samples with stem cells presented a more differentiated gray level than the 10-week samples without stem cells ( ***Fig. 1***).

### Histological analysis results

**Fig.2:**
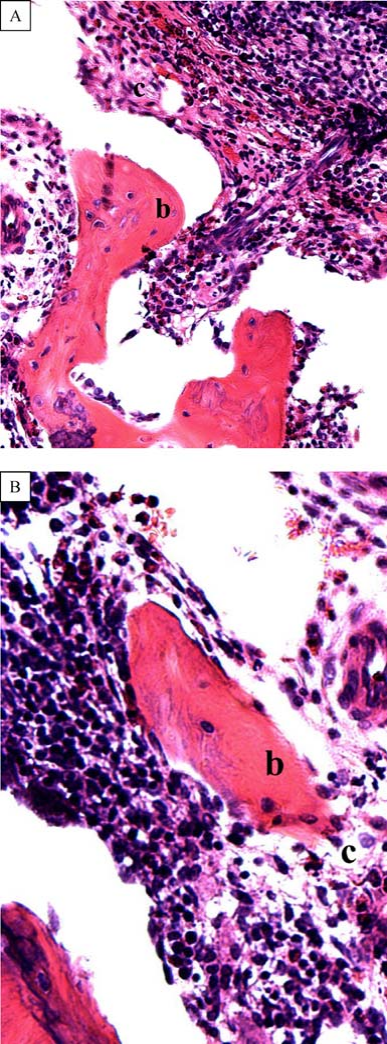
**Haematoxylin-eosin staining, at two-week sample.** A: Bio-Oss® Block without stem cells (×100); B: Bio-Oss® Block with stem cells). Note the bone tissue (b) and connective tissue (c) (×200).

Specimens at 2 weeks: There were no inflammatory cells around the particles in animals treated with deproteinized bovine bone material associated with mesenchymal stem cells. Most particles were surrounded by newly formed connective tissue. In some areas, newly formed bone matrix was firmly adhering to the surface of the deproteinized bovine bone, with no spaces at the interface. In other areas, the islands of newly formed connective tissue were not closely related to the particles of deproteinized bovine bone material. In these cases, the surface was almost entirely covered by rounded cells that appeared to be active and wrinkled by a dense extracellular matrix. The findings of the left side defects were characterized by the presence of filler material with connective tissue around it. Deproteinized bovine bone units were easily distinguishable from newly formed connective tissue in the samples ( ***Fig. 2***).

**Fig.3:**
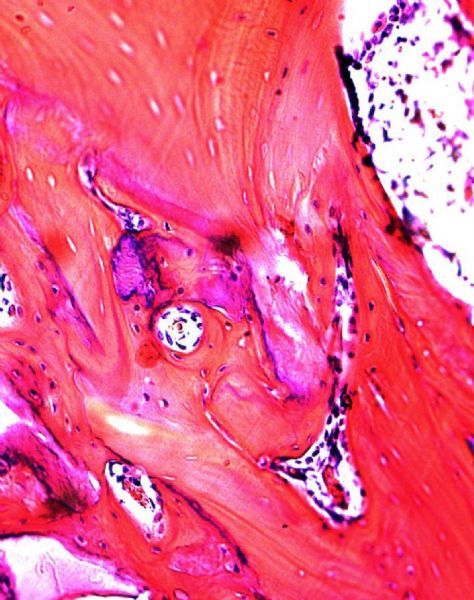
**Newly bone tissue.** The Haversian canal is located in the center of the image (×100).

**Fig.4:**
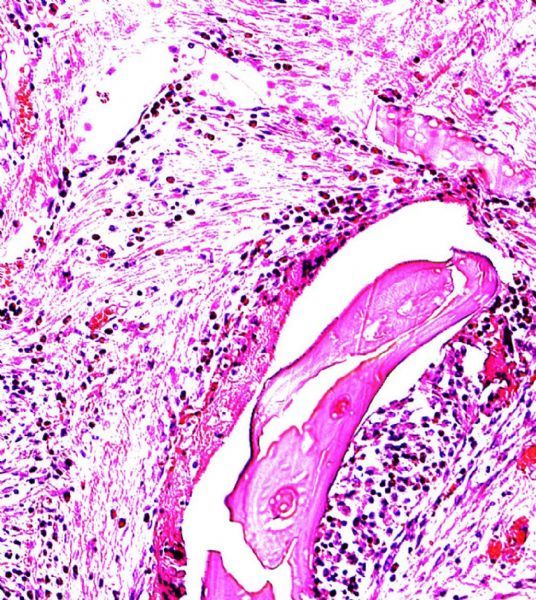
At 4 weeks Bio-Oss Collagen ® with stem cells (×100).

Specimens at 4 weeks: The histological study of the side shows areas of connective tissue with clearly distinguishable areas of newly formed bone tissue. In particular, bone tissue presented a typical structure of bone tissue such as lamellar histological structure ( ***Fig. 3***). Moreover, scaffold particles were still distinguishable from newly formed bone tissue and connective tissue. There was no inflammatory infiltrate at the interface or around the particles. In the control side, histology was consistent with that shown at 4 weeks ( ***Fig. 4***).

**Fig.5:**
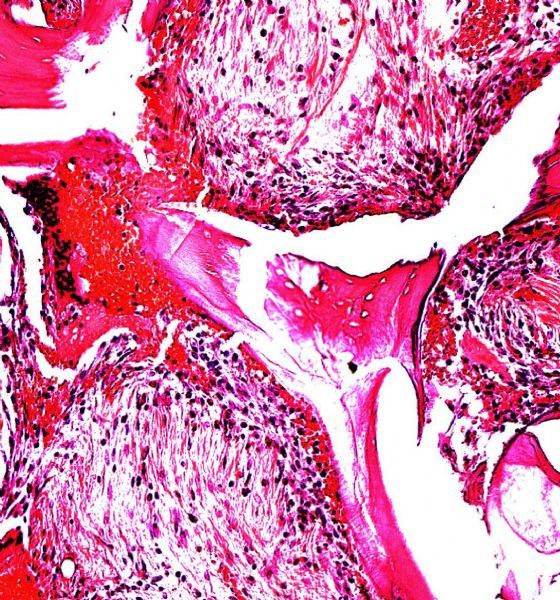
Hematoxylin-eosin staining, showing the Bio-Oss® Block without stem cells at 6 weeks (×100).

**Fig.6:**
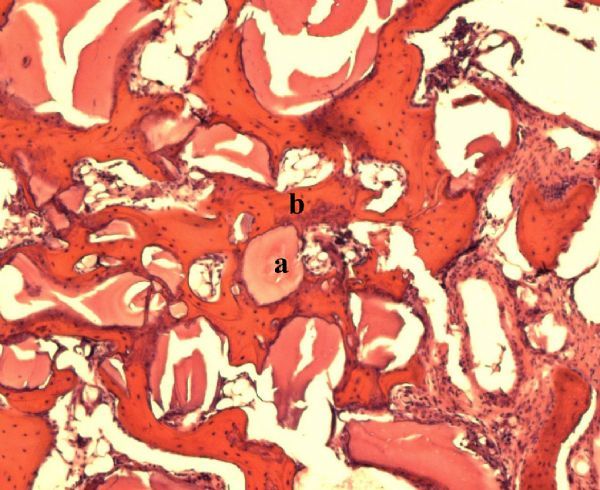
At 6 weeks Bio-Oss® Block (a) with stem cells and bone tissue (b) (×100).

Specimens at 6 weeks: In the test side, particles of scaffold surrounded by maturing bone were easily visible ( ***Fig. 5*** and ***6***).

Specimens at 10 weeks: In samples of last sacrificed animals complete ossification, demonstrated by trabecular bone structure still maturing, absence of connective tissue, completely replaced by bone in every regenerated defects. Only in the empty defect we can observe trabecular bone still surrounded by islands of connective tissue.

As a very important result, histology confirmed the formation of the ossification front already deducible from micro-CT analysis. This ossification front was a zone of lower density of both healthy bone and scaffold, situated at the interface and growing with the temporal distance between the implant and animal sacrifice ( ***Fig. 6 ***and ***7***).

**Fig.7:**
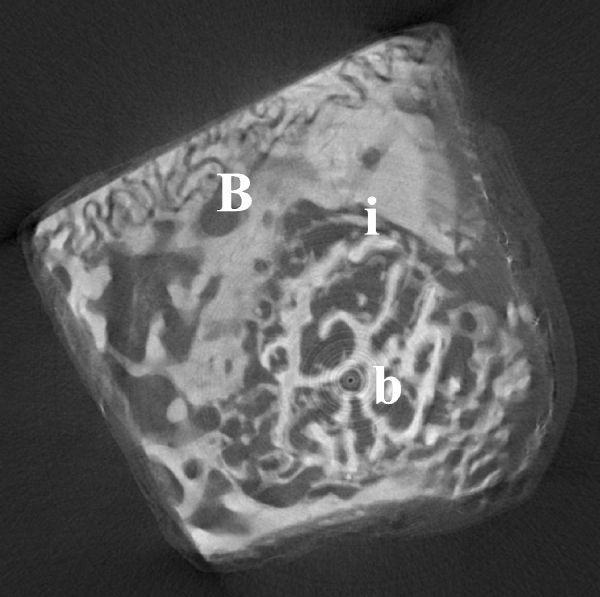
**Micro-CT image of an intraosseus defect with Bio-Oss® Block and stem cells at 10 weeks. ** Note the interface (i) between bone (b) and biomaterial (B).

There seems to be no significant differences in regeneration between Bio-Oss ® Block and Bio-Oss Collagen®. Both scaffolds were not easy to handle: Bio-Oss ® Block must be specially modified in order to adapt to the defect. Its solid structure was optimal to support the overlying tissues and to provide the needed support for the bone to regenerate. On the contrary, Bio-Oss Collagen® instead had a spongiform structure losing most of its mechanical ability in contact with the blood. It was also not indicated for supporting tissues. Nevertheless, it is extremely easy to handle by non expert operators and it is an excellent medium for growing the bone.

## Discussion

Eight New Zealand rabbits were used for this study. After surgery and up to the date of sacrifice, the recovery of all the animals involved in this study was considered normal in terms of eating behavior, weight and quality of life. To the best of our knowledge, in the present literature, there is no evidence of a general agreement on the correct animal model to be used for scientific purposes similar to the ones involved in this study. Some authors claim that one should use a model in which bone biology and composition is very similar to that of humans ( *i.e.* dog, sheep, goat, pig or monkey)^[20]^. Indeed, they claim that the trabecular bone contained in rodent bones is poor also in the metaphysis of long bones and that re-modeling of Haversians channels by osteoclastic re-absorption does not happen in rodents^[21]^. On the contrary, other authors, without denying the previous observations, claim that the use of the above mentioned animal models present some drawbacks^[2]^. Rodents are the most common animals employed during *in vivo* studies reported in the literature; this is mainly related to practical reasons: large animals are more expensive, time consuming and more difficult to keep.

There are multiple factors that affect osseous bone healing that are best evaluated in the *in vivo* environment, including biomechanical, cellular and vascular mechanics that comprise the healing process. The model chosen to assess a particular device should mimic the environment in which the device will be used therapeutically. One should choose a “critical-size defect” that will not spontaneously regenerate, to best characterize the contribution of the device to healing. Tissue engineering devices can be screened in various preclinical animal models to determine their potential. Depending on previous data on a product, one can choose the appropriate model to screen the potential of a new device. For new technology, it is advised to begin with small animal models, which can provide early data in a relatively fast and cost-effective way. This kind of research can progress with systems that simulate the human wound and therapeutic environment more closely in association with the planned clinical application of the device^[22]^.

Both scaffolds exhibit open-pore structures allowing cell penetration and attachment^[23–25]^. Numerous studies have employed defects in the calvarium as the site in which to screen biomaterials for the bone response that they elicit, principally because the diameter of the critical-size defect is smaller than that in long bone sites. Another advantage is that multiple defects can be produced in the same surgical field. An incision is made through the scalp to expose the periosteum, which is then elevated and retracted to expose the bone. Defects can be produced with standardized trephine burs, using continuous saline irrigation for cooling. The biomaterial can then be placed into the defects^[26]^.

Having shown no difference between two scaffold-groups regarding bone regeneration, micro-CT and histological examination revealed the presence of early bone changes.

Modern techniques of bone regeneration are still very far from satisfactorily resolving all situations in which they are needed. Considerable help in the future could come from Tissue Engineering. This last can provide new and "targeted" tools enabling tissue-regenerating stimuli to arrive at the aimed cellular lines only. The new bone, formed with adipose derived stem cells, seems to be superposable with that obtained by traditional regenerative technique, both in the histological appearance and in the timing of neodeposition and calcification of the extracellular matrix. Further studies should be conducted in order to understand what the contribution is to regeneration of adult stem cells. The engineering of biomaterials plays a major role in this field. Indeed, the use of selected and differentiated cells should be combined with a specially designed scaffold, allowing amplifying the cells potential. Currently, there is no available scaffold meeting all the requirements of tissue engineering and research should be focused in this direction.
